# CD38 promotes angiotensin II‐induced cardiac hypertrophy

**DOI:** 10.1111/jcmm.13076

**Published:** 2017-03-12

**Authors:** Xiao‐Hui Guan, Xuan Hong, Ning Zhao, Xiao‐Hong Liu, Yun‐Fei Xiao, Ting‐Tao Chen, Li‐Bin Deng, Xiao‐Lei Wang, Jian‐Bin Wang, Guang‐Ju Ji, Mingui Fu, Ke‐Yu Deng, Hong‐Bo Xin

**Affiliations:** ^1^ Institute of Translational Medicine Nanchang University Nanchang China; ^2^ National Laboratory of Biomacromolecules Institute of Biophysics Chinese Academy of Sciences Beijing China; ^3^ Department of Basic Medical Science Shock/Trauma Research Center School of Medicine University of Missouri Kansas City Kansas City MO USA

**Keywords:** CD38, cardiac hypertrophy, SIRT3, angiotensin II, NFATc4

## Abstract

Cardiac hypertrophy is an early hallmark during the clinical course of heart failure and regulated by various signalling pathways. Recently, we observed that mouse embryonic fibroblasts from CD38 knockout mice were significantly resistant to oxidative stress such as H_2_O_2_‐induced injury and hypoxia/reoxygenation‐induced injury. In addition, we also found that CD38 knockout mice protected heart from ischaemia reperfusion injury through activating SIRT1/FOXOs‐mediated antioxidative stress pathway. However, the role of CD38 in cardiac hypertrophy is not explored. Here, we investigated the roles and mechanisms of CD38 in angiotensin II (Ang‐II)‐induced cardiac hypertrophy. Following 14 days of Ang‐II infusion with osmotic mini‐pumps, a comparable hypertension was generated in both of CD38 knockout and wild‐type mice. However, the cardiac hypertrophy and fibrosis were much more severe in wild‐type mice compared with CD38 knockout mice. Consistently, RNAi‐induced knockdown of CD38 decreased the gene expressions of atrial natriuretic factor (ANF) and brain natriuretic peptide (BNP) and reactive oxygen species generation in Ang‐II‐stimulated H9c2 cells. In addition, the expression of SIRT3 was elevated in CD38 knockdown H9c2 cells, in which SIRT3 may further activate the FOXO3 antioxidant pathway. The intracellular Ca^2+^ release induced by Ang‐II markedly decreased in CD38 knockdown H9c2 cells, which might be associated with the decrease of nuclear translocation of NFATc4 and inhibition of ERK/AKT phosphorylation. We concluded that CD38 plays an essential role in cardiac hypertrophy probably *via* inhibition of SIRT3 expression and activation of Ca^2+^‐NFAT signalling pathway. Thus, CD38 may be a novel target for treating cardiac hypertrophy.

## Introduction

Cardiac hypertrophy, an abnormal enlargement of the heart muscle, is often associated with increased afterloading of heart. It may be physiological, as occurs in athletes, or pathological, due to underlying cardiac disease such as congestive heart failure, valve disease, hypertension 1, 2. It has been reported that development of cardiac hypertrophy may be involved in multiple signalling pathways such as the canonical G‐protein‐coupled receptors (GPCR) signalling pathway, the calcineurin–nuclear factor of activated T cells (NFAT) signalling pathway, the mitogen‐activated protein kinase (MAPK) signalling pathway and PI3K/Akt/GSK‐3β signalling pathway 3. In general, blocking one or some of the hypertrophic signalling pathways could alleviate or even stop the progress of cardiac hypertrophy.

CD38, a 45‐kD and a type II transmembrane glycoprotein, comprises a short N‐terminal cytoplasmic domain, a hydrophobic transmembrane region and a long C‐terminal extracellular domain 4. It is first identified as a leucocyte differentiation antigen 5 and ubiquitously distributed in lymphoid tissues and non‐lymphoid tissues such as brain, eye, pancreas, prostate, gut and muscle 6. It is also a multifunctional enzyme that possesses both ADP‐ribosyl cyclase and cyclic ADP ribose (cADPR) hydrolase activities, by which it is able to cleave NAD^+^ to cADPR and hydrolyse cADPR to ADP ribose (ADPR) 7. As a major NADase of mammalian cells, CD38 plays an important role in maintaining intracellular NAD^+^ levels. Previous evidence indicates that CD38 deficiency remarkably elevates the intracellular NAD^+^ levels in multiple tissues 8. Among all the stimuli that lead to the development of cardiac hypertrophy, oxidative stress is one of the key stimuli 9. Oxidative stress is always associated with NAD^+^ depletion 10 or NAD^+^‐consuming enzymes activation. Low level of intracellular NAD^+^ could make a cell unable to defend against oxidative stress for limiting its energy‐dependent functions, which are supported by some NAD^+^‐dependent enzyme such as sirtuins.

Sirtuins are class III HDACs involved in gene silencing, differentiation, cell survival, metabolism and longevity 11. There are seven sirtuins (SIRT1‐7) in mammalian cells, and they are distributed in different subcellular compartments, including the nucleus (SIRT1, SIRT2, SIRT6 and SIRT7), cytoplasm (SIRT1 and SIRT2) and mitochondria (SIRT3, SIRT4 and SIRT5) 12. SIRT1, a member of the family which is well studied, has already been reported to play a protective role against ageing and age‐related diseases, such as cardiovascular disease, neurodegenerative disease, osteoporosis, chronic kidney disease and metabolic syndrome 11. It is also reported that SIRT3 could protect myocytes from oxidative stress‐mediated cell death through elevating expression of antioxidants 13 and is the only one implicated in longevity in humans 14. Recently, Pillai *et al*. demonstrated that SIRT3 is highly expressed in cardiomyocytes and may protect the cells from Ang‐II‐induced cardiac hypertrophy through exogenous NAD^+^‐mediated activation of the SIRT3–LKB1–AMPK pathway 15. Interestingly, disruption of CD38 gene could elevate intracellular NAD^+^ level as supplying with exogenous NAD^+^. More recently, we found that mouse embryonic fibroblasts (MEFs) from CD38 KO mice were significantly resistant to oxidative stress such as H_2_O_2_‐induced injury and hypoxia/reoxygenation (H/R)‐induced injury compared with wild‐type MEFs 6. We also observed that CD38 deficiency protected heart of the mice from ischaemia reperfusion injury through activating SIRT1/FOXO3 mediated an antioxidative stress pathway 16. Therefore, we hypothesize that CD38 may play an essential role in Ang‐II‐induced cardiac hypertrophy.

Ca^2+^–calcineurin–NFAT signalling is a classic pathway that induces cardiac hypertrophy 17. Numerous studies have reported that cADPR, an important Ca^2+^ mobilizer, triggers IP3‐independent Ca^2+^ release from sarcoplasmic reticulum like acetylcholine 18, oxotremorine 19, Ang‐II 20 and endothelin 21. Notably, calcineurin–NFAT signalling pathway is also interacted with other hypertrophic pathways, such as MAPK, GSK3, p38 and JNK 22.

Therefore, this study aimed to examine the role of CD38 in Ang‐II‐induced cardiac hypertrophy and to elucidate the mechanisms by which CD38 regulates the hypertrophic process. In this study, we have examined the effects of CD38 deficiency on the development of cardiac hypertrophy. The results showed that CD38 is a positive regulator of cardiac hypertrophy *via* inhibition of SIRT3–FOXO3 pathway and activation of Ca^2+^–calcineurin–NFAT signalling. Thus, inhibition of CD38 might represent a novel strategy for curing pathological cardiac hypertrophy.

## Materials and methods

### Mice and animal model

CD38 knockout mice were obtained from Dr. Frances E. Lund (University of Rochester, USA) and generated by Professor Maureen C. Howard 23. Ten‐ to twelve‐week‐old male wild‐type and CD38 knockout mice were randomly assigned to the control or Ang‐II‐treated group, which were infused with saline or Ang‐II (1500 ng/kg/day) for 14 days by implanting osmotic mini‐pumps (ALZET model 2002) in the peritoneal cavity, respectively. Elevation of systolic blood pressure was measured by tail‐cuff system (softron BP‐98A) and used as an indicator for examining the efficiency of the mouse model. The Vevo770 High‐Resolution Imaging System was used for the echocardiography analysis. All measurements were covered for at least five consecutive cardiac cycles. The animals were handled according to the animal welfare regulation of Nanchang University, Nanchang, China, and the study protocol was approved by the animal subjects committee of Nanchang University.

### Histology

Heart tissues were fixed in 4% paraformaldehyde, embedded in paraffin and sectioned at 3‐μm intervals. Masson trichrome staining was performed according to the instruction manual. Picrosirius red staining was used for examining the cardiac fibrosis area, and the collagen types I and III were differentiated by a polarizer. A 3‐μm section was de‐paraffinized and incubated with 100 μg/ml FITC‐labelled wheat germ agglutinin (Sigma‐Aldrich, St. Louis, MO) for 30 min. at 37°C. Sections were viewed with an Olympus IX71 inverted microscope.

### Cell culture and transfection

H9c2 cells (ATCC, CRL‐1446™) were cultured in DMEM (Thermofisher, Waltham, MA, USA) supplemented with 10% FBS and 100 μg/ml of each of penicillin and streptomycin (Thermofisher) at 37°C with 5% CO_2_. The CD38 knockdown H9c2 stable cell line was prepared as we described previously 16. In different experiments, different cell numbers were used. The cells used for RNA and total protein detection were seeded at 5 × 10^5^ cells/3.5 cm dish for 48 hrs Ang‐II treatment, while only 1 × 10^5^ cells/3.5 cm dish were seeded for Crystal violet staining. In the experiments that need only 20‐min. Ang‐II treatment, 1 × 10^6^ cells/3.5 cm dish were used.

### ROS detection

The intracellular ROS contents were measured by staining with 2,7‐dihydrodichlorofluorescein diacetate (H2DCF‐DA; Sigma‐Aldrich) and monitoring the fluorescent dichlorofluorescein. Cells were incubated with 10 μM H2DCF‐DA for 20 min. Then, the cells were washed twice with PBS and collected for flow cytometry assay. Five thousand cells were analysed for each sample. All the steps should be kept away from light.

### Real‐time quantitative PCR

Total RNAs were extracted with TRIzol (Invitrogen, Carlsbad, CA, USA) and subjected to cDNA synthesis using M‐MLV reverse transcriptase kit (Invitrogen). Gene expression was analysed by real‐time quantitative reverse transcription polymerase chain reaction according to the instructions (Invitrogen) and normalized by the reference gene GAPDH. The sequences of the primers are as follows: GAPDH‐forward primer 5′‐AGCCAAAAGGGTCATCATCT‐3′, GAPDH‐Reverse primer 5′‐GGGGCCATCCACAGTCTTCT‐3′; ANP‐forward primer 5′‐ATCTGATGGATTTCAAGAACC‐3′, ANP‐reverse primer 5′‐CTCTGAGACGGGTTGACTTC‐3′; BNP‐forward primer 5′‐ACAATCCACGATGCAGAAGCT‐3′, BNP‐reverse primer 5′‐GGGCCTTGGTCCTTTGAGA; FOXO3‐forward primer 5′‐CCTGTCCTACGCTGACCTGAT‐3′, FOXO3‐reverse primer 5′‐AGTCCCTTCGTTCTGAACCCG‐3′; ARC‐forward primer 5′‐CGGAAACGGCTGGTAGAAAC‐3′, ARC‐reverse primer 5′‐AGTCCCTTCGTTCTGAACCCG‐3′; Bcl2‐forward primer 5′‐GTTGCAGTCACCGGATTCCT‐3′, Bcl2‐reverse primer 5′‐CGGA GGTGGTGTGAATCCA‐3′; SOD2‐forward primer 5′‐GGACAAACCTGAGCCCTAA‐3′, SOD2‐reverse primer 5′‐GCGACCTTGCTCCTTATTG‐3′.

### Western blotting analysis

Cells were lysed using RIPA buffer with proteinase cocktail (Roche Diagnostics, Mannheim, Germany). Proteins were separated by 10% SDS‐PAGE and transferred to PVDF membrane. The membrane was blocked with 5% non‐fat milk for 60 min. and incubated with the primary antibody at 4°C overnight. The antibodies of CD38 (Santa Cruz sc‐7049), AKT (CST), p‐AKT (CST), ERK (CST), p‐ERK (CST), GSK3β (CST), p‐GSK3β (CST) and SIRT3 antibody (CST) were used at 1:1000 dilution, respectively. The membrane was then incubated with HRP‐conjugated secondary antibody at 1:5000 dilutions for 1 hr at room temperature, and the immune complexes were visualized by enhanced chemiluminescence method. The nuclear and cytoplasmic extraction was performed with the NE‐PER nuclear and cytoplasmic extraction reagents (Thermo fisher) according to the operation manual.

### Calcium assay

After 20‐min. Ang‐II treatment, cells were incubated with 5 μM Fluo3‐AM (Sigma‐Aldrich) at 37°C for 30 min.; then, the cells were washed several times with PBS and further incubated for 40 min. Fluorescent was detected using an inverted microscope.

### Data analysis

Student's paired two‐tailed test was applied to determine statistical significance between two groups. Differences were considered to be statistically significant when *P* value was less than 0.05.

## Results

### CD38 deficiency prevented mice from Ang‐II‐induced cardiac hypertrophy *in vivo*

**Figure 1:**
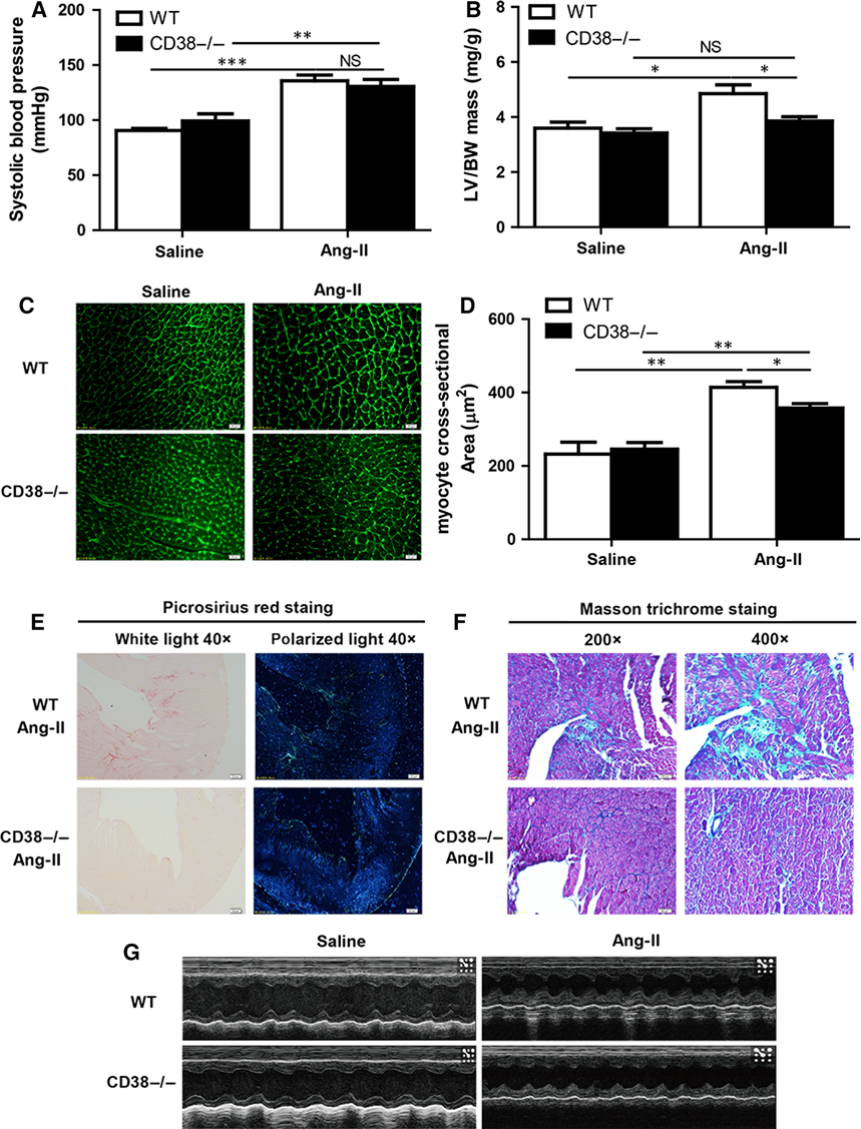
CD38 deficiency prevents mice from Ang‐II‐induced cardiac hypertrophy. (**A**) Alternations of blood pressure by Ang‐II infusion in mice. The systolic blood pressure (mmHg) was measured from wild‐type (WT) and CD38 knockout (CD38^−/−^) mice with or without 14 days of Ang‐II infusion (*n* = 5, **P < 0.01, ***P < 0.001). (**B**) Quantitative analysis of left ventricular mass/body weight (LV/BW) ratio. The left ventricular mass was measured by echocardiography, and the LV/BW ratio was calculated from both WT and CD38^−/−^ mice after Ang‐II infusion (*n* = 5, *P < 0.05). (**C**) Wheat germ agglutinin staining of cardiomyocytes. The cardiomyocytes were stained by Wheat germ agglutinin, and the cell sizes in heart sections were measured after Ang‐II infusion (magnification, ×400, scale bars, 20 μm). (**D**) Quantitative analysis of cardiomyocyte cross‐sectional area. The 200 cells per heart were quantitatively measured in CD38^−/−^ mice compared with wild‐type mice with or without Ang‐II stimulation (*n* = 3,*P < 0.05, **P < 0.01,***P < 0.001). (**E**) Picrosirius red staining of collagen type I and III. The collagen type I (orange) and III (green) contents of WT and CD38^−/−^ hearts after 14 days of saline or Ang‐II infusion were stained by Picrosirius red under light microscopy (left, magnification, ×40, scale bars, 200 μm) and polarized with light microscopy (right, magnification, ×40, scale bars, 200 μm). (**F**) Masson staining of collagen deposition. The collagen deposition in the hearts was stained by Masson trichrome after 14 days saline or Ang‐II infusion. Masson trichrome‐stained area was in light blue (magnification ×200, scale bars, 50 μm; magnification ×400, scale bars, 20 μm). (**G**) A typical echocardiography of M‐mode. The echocardiographies of the left ventricle with M‐mode were measured from WT and CD38^−/−^ mice with 14 days of saline or Ang‐II infusion (NS indicates not significant. *n* = 5, *P < 0.05, **P < 0.01, ***P < 0.001).

Western blot analysis was first used to confirm the protein expression of CD38 in wild‐type and CD38 KO mice (Fig. S1). To observe the role of CD38 in cardiac hypertrophy, both CD38 knockout and wild‐type mice were subjected to Ang‐II infusion for 14 days. The results showed that the systolic blood pressures were markedly increased in both mouse models after Ang‐II infusion (Fig. 1A), indicating that the hypertension mouse models were successfully induced. Meanwhile, there was no difference in the blood pressures between the WT and CD38‐deficient mice, suggesting that CD38 did not affect Ang‐II‐induced hypertension. Ang‐II infusion significantly increased the ratio of left ventricle/body weight (LV/BW) by almost 24% in wild‐type mice, but not in CD38‐deficient mice (Fig. 1B), indicating that CD38 was required for Ang‐II‐induced cardiac hypertrophy. To assess the role of CD38 in regulating cardiac hypertrophy, we used wheat germ agglutinin staining to evaluate cardiomyocyte size. The cardiomyocyte size for WT and CD38^−/−^ mice was comparable after saline infusion (Fig. 1C), whereas Ang‐II infusion significantly increased the cell size of WT mice, in which the cardiomyocyte size of CD38^−/−^ mice was attenuated by 12% compared with that of wild‐type mice (Fig. 1D). In addition, to determine whether CD38 deficiency also affects Ang‐II‐induced cardiac remodelling, we examined cardiac fibrosis of WT and CD38^−/−^ hearts after Ang‐II infusion. After 2 weeks of Ang‐II infusion, collagen deposition was significantly increased in LV tissues of WT hearts, particularly in the interstitial and perivascular areas. In contrast, the cardiac fibrosis of CD38^−/−^ mice was significantly attenuated compared with wild‐type mice after Ang‐II infusion. Picrosirius red staining was used for analysis of total collagen contents on bright field microscopy, and collagen fibres were detected by polarized light microscopy. The contents of Collagen I and III fibres were lower in CD38^−/−^ than that of WT hearts after Ang‐II infusion (Fig. 1E and Fig. S2). As showed in Figure 1F and Figure S3, the collagen accumulation tested by Masson trichrome staining was greater in the interstitial and perivascular sites in WT hearts than CD38^−/−^ hearts after Ang‐II infusion.

To examine the effect of CD38 on cardiac function after Ang‐II infusion, echocardiography was measured for each mouse. The M‐mode echocardiography of the left ventricle in WT and CD38^−/−^ mice with saline or Ang‐II infusion was showed in Figure 1G. The results showed that posterior wall thickness and LV mass were smaller in CD38^−/−^ hearts than that in WT hearts, but there were no remarkable changes for the ejection fraction (EF) and fractional shortening (FS) (Table 1). Thus, our results indicated that CD38 deficiency attenuated Ang‐II‐induced cardiac hypertrophy but not affect the cardiac functions.

**Table 1:** Echocardiography in Ang‐II‐infused WT and CD38^−/−^ mice

Parameter	WT Saline	CD38^−/−^ Saline	WT Ang‐II	CD38^−/−^ Ang‐II
LVPW d, mm	0.876 ± 0.087	0.817 ± 0.041	1.387 ± 0.053	1.087 ± 0.090*
LVAW d, mm	0.898 ± 0.060	0.836 ± 0.043	0.980 ± 0.088	1.037 ± 0.033
LVlD d, mm	3.351 ± 0.195	3.506 ± 0.093	3.045 ± 0.275	2.805 ± 0.094
LVPW s, mm	1.227 ± 0.065	1.225 ± 0.082	1.817 ± 0.091	1.557 ± 0.078*
LVAW s, mm	1.353 ± 0.078	1.356 ± 0.044	1.505 ± 0.042	1.484 ± 0.086
LVlD s, mm	2.253 ± 0.127	2.230 ± 0.066	1.744 ± 0.170	1.742 ± 0.186
EF, %	64.23 ± 1.656	63.134 ± 1.478	78.394 ± 4.382	74.014 ± 5.955
FS, %	34.129 ± 1.210	33.388 ± 1.167	46.453 ± 3.936	42.886 ± 5.114
LV Mass, mg	81.752 ± 7.398	79.517 ± 3.635	110.602 ± 11.822	82.151 ± 3.766

EF: ejection fraction; FS: fractional shortening; LV mass: left ventricular mass; LVAWd: left ventricular anterior wall diastolic thickness; LVAWs: left ventricular anterior wall systolic thickness; LVIDd: left ventricular internal diastolic dimension; LVIDs: left ventricular internal systolic dimension; LVPWd: left ventricular posterior wall diastolic thickness; LVPWs: left ventricular posterior wall systolic thickness. Data are mean ± S.E.M., *n* = 5, *P < 0.05 *versus* Ang‐II infused WT mice.

### RNAi‐mediated knockdown of CD38 prevented Ang‐II‐induced cardiac hypertrophy *in vitro*

**Figure 2:**
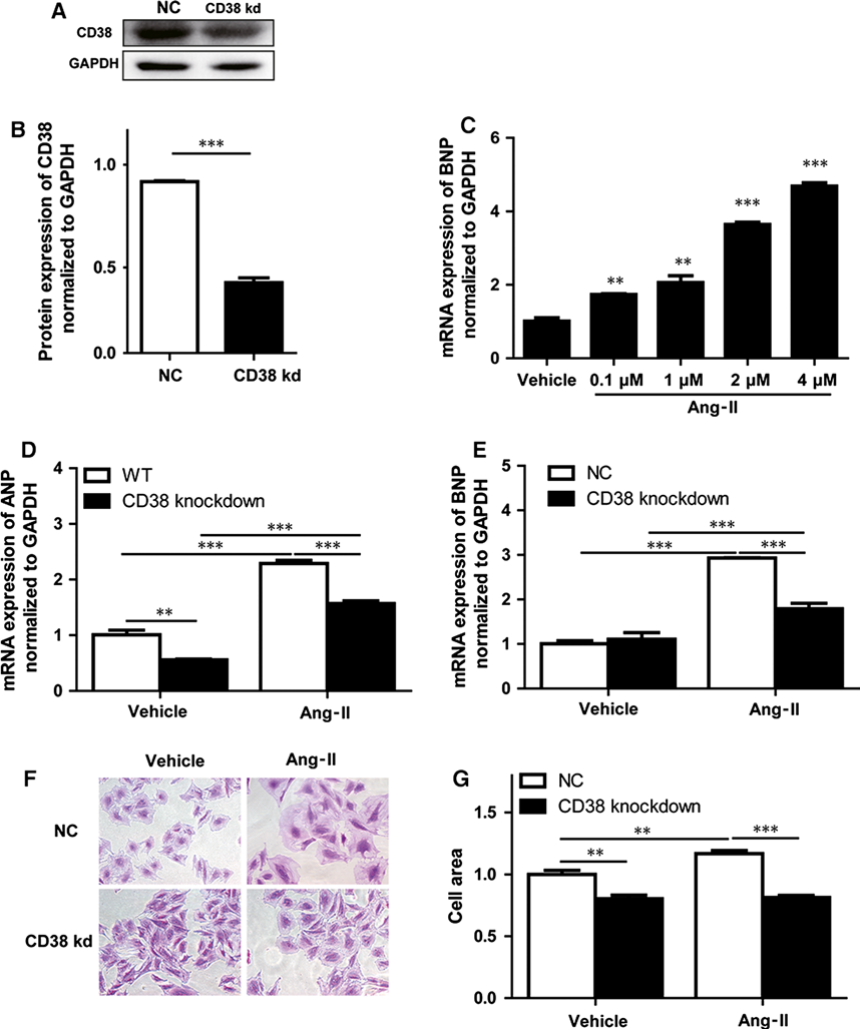
CD38 deficiency attenuated Ang‐II‐induced cardiac hypertrophy *in vitro*. (**A** and **B**) CD38 expressions in CD38 knockdown stable cell line after Ang‐II treatment. The CD38 expressions were analysed by Western blot in wild‐type or CD38 knockdown stable cell lines (**A**), and the protein expressions were quantified from at least three different experiments and GAPDH as an internal control (**B**). (**C**) The mRNA expression levels of BNP induced by Ang‐II stimulation. The BNP mRNA expressions were detected by quantitative RT‐PCR with H9c2 cells after 48 hrs stimulation of different concentrations of Ang‐II. (**D**) The mRNA expression levels of ANP induced by Ang‐II stimulation. The ANP mRNA expressions were detected by quantitative RT‐PCR with CD38 knockdown or wild‐type H9c2 cells after 48 hrs stimulation of 2 μM Ang‐II. (**E**) The mRNA expression levels of BNP induced by Ang‐II stimulation. The BNP mRNA expressions were detected by quantitative RT‐PCR in CD38 knockdown or wild‐type H9c2 cells after 48 hrs stimulation of 2 μM Ang‐II. (**F** and **G**) Crystal violet staining of H9c2 cells. The H9c2 cells including CD38 knockdown or wild‐type cells were stained with crystal violet (magnification, ×200 scale bars, 50 μm; magnification, ×400 scale bars, 20 μm) after stimulation with 2 μM Ang‐II for 48 hrs (**F**), and the cell areas were quantitatively analysed from four independent experiments. *N* = 4, **P < 0.01, ***P < 0.001.

To confirm the protective role of CD38 deficiency in Ang‐II‐induced cardiac hypertrophy, the stable CD38 knockdown H9c2 cell lines were prepared with CD38‐specific shRNA construct and the reduced expression of CD38 in the cell lines was confirmed by Western blot (Fig. 2A and B). The stable cell lines were subjected to 48 hrs Ang‐II stimulation, as we expected, the mRNA expression of BNP was up‐regulated by Ang‐II in a dose‐dependent manner (Fig. 2C). Ang‐II significantly increased the expression of ANP and BNP. However, knockdown of CD38 significantly decreased Ang‐II‐induced expressions of ANP and BNP which serve as the markers of cardiac hypertrophy (Fig. 2D and E). Next, H9c2 cells were stained with crystal violet. As shown in Figure 2F and G, the cardiomyocyte sizes induced by Ang‐II were much larger than that of the untreated cells in normal cell lines, whereas CD38 knockdown cells were not enlarged after Ang‐II stimulation.

### CD38 knockdown attenuated Ang‐II‐induced oxidative stress in H9c2 cell lines

**Figure 3:**
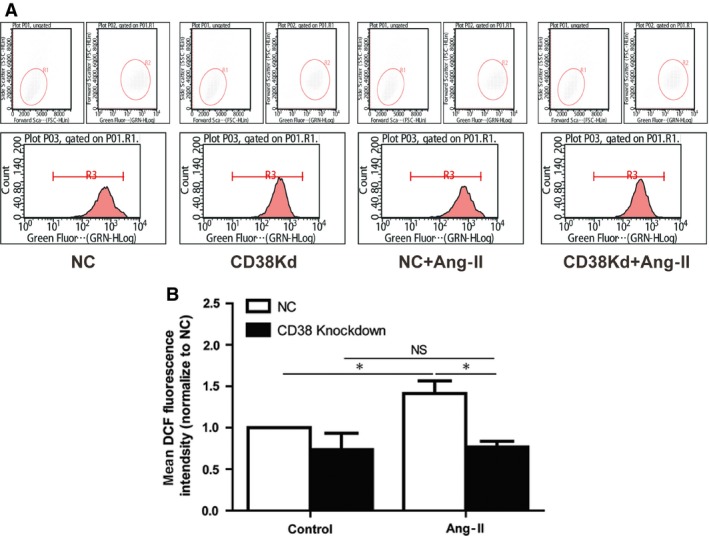
Knockdown of CD38 decreased Ang‐II‐induced ROS generation. (**A**) Measurements of ROS productions. Negative control and CD38 gene knockdown H9c2 cells were stained with CM‐H2DCFDA, and the ROS production was measured by fluorescence‐activated cell sorter after the cells were subjected to 20 min. Ang‐II stimulation. (**B**) Quantification analysis of the mean fluorescence intensity. The Ang‐II‐induced ROS productions were quantitatively analysed with different groups of cells (*n* = 3, *P < 0.05).

Mounting evidence indicates that induction of ROS is necessary for the development of cardiac hypertrophy 24. To examine whether CD38 also affect Ang‐II‐induced production of ROS, H9c2 cells were treated by Ang‐II for 20 min., and then stained with CM‐H_2_DCFDA, a non‐fluorescent dye that fluoresced by ROS‐mediated removal of an acetated group by intracellular esterases. We found that ROS generation induced by Ang‐II was attenuated by CD38 knockdown (Fig. 3A and B), indicating that CD38 may be involved in the production of ROS.

### Knockdown of CD38 inhibited Ang‐II‐induced hypertrophy through activating of SIRT3‐FOXO3 signalling pathway

**Figure 4:**
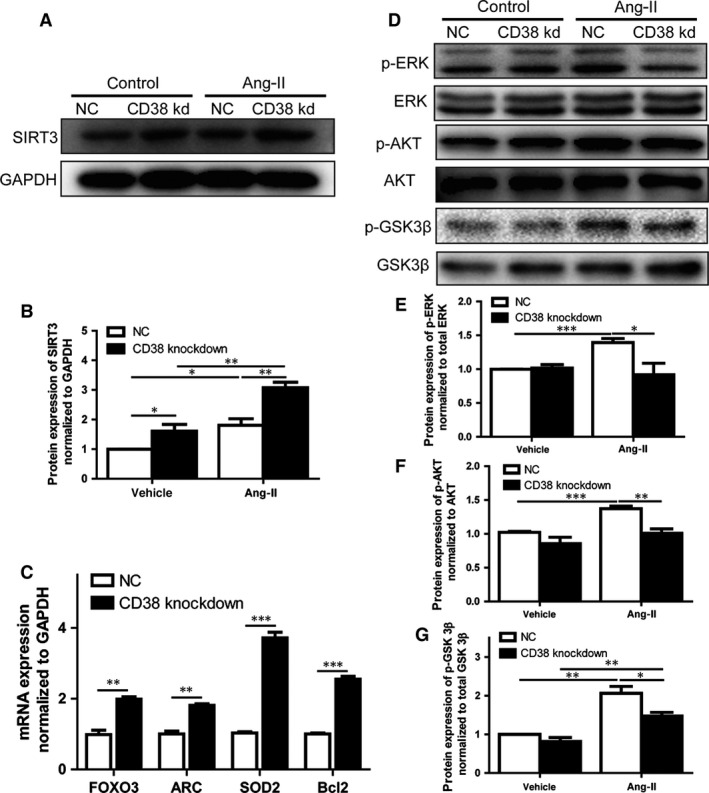
Effects of CD38 knockdown on the activation of SIRT3‐FOXO3 signalling **pathway** induced by Ang‐II stimulation. (**A** and **B**) Effects of CD38 knockdown on Sirt3 expressions in H9c2 cells. The expressions of SIRT3 were determined by Western blot in CD38 knockdown or wild‐type H9c2 cells with or without the stimulation of Ang‐II for 48 hrs (**A**), and the quantitative analysis was also performed (**B**). (**C**) The alternations of mRNA expressions of FOXO3, ARC, Bcl2 and SOD2. The mRNA expressions of these genes were examined by qRT‐PCR in CD38 knockdown or control H9c2 cells. (**D–G**) Effects of CD38 knockdown on ERK, ATK and GSK3β. The protein expressions of total and phosphorylated ERK/AKT/GSK3β were detected by Western blot after 20 min. stimulation of Ang‐II (**D**). The phosphorylations of ERK (**E**), AKT (**F**) and GSK3β (**G**) were quantitatively determined using corresponding phosphorylation‐specific antibodies. The experiments were repeated at least three times. *N* = 4, *P < 0.05, **P < 0.01, *P < 0.001.

As a major NADase in mammalian cells, CD38 cleaves NAD^+^ to cADPR. It has been reported that deficiency of CD38 could elevate intracellular NAD^+^ level 8, 25. Previous studies showed that increased intracellular NAD^+^ level up‐regulated the expression of sirtuins, including SIRT1 and SIRT3 26. Moreover, exogenous NAD^+^ may suppress cardiac hypertrophy *via* activation of SIRT3–LKB1–AMPK signalling pathway 15. Accordingly, we examined the protein expressions of SIRT3 after Ang‐II stimulation. As expected, knockdown of CD38 increased SIRT3 expressions in H9c2 cells and Ang‐II further increased SIRT3 expression levels in the CD38 knockdown cells compared with normal H9c2 cells (Fig. 4A and B). It has been reported that SIRT3, a stress‐responsive deacetylase, blocks the cardiac hypertrophic response through activating FOXO‐dependent antioxidants, manganese superoxide dismutase and catalase, as well as suppressing ROS‐mediated Ras activation and its downstream signalling pathways such as MAPK/ERK and PI3K/Akt pathways 26. Our results showed that CD38 knockdown promoted the mRNA expressions of FOXO3 and increased the expressions of its downstream molecules ARC, Bcl2 and SOD2 as showed in Figure 4C, indicating that CD38 gene silence indeed enhances the expression and transcriptional activity of FOXO3. After 20‐min. Ang‐II stimulation, we also observed that the phosphorylations of AKT1, ERK and GSK3β were increased in normal H9c2 cells, but not in CD38 knockdown H9c2 cells (Fig. 4D–G). All the results suggested that CD38 gene silence protects heart from hypertrophy through activating SIRT3–FOXO3‐mediated antioxidative signalling pathway, and suppressing ROS‐mediated MAPK/ERK and PI3K/Akt signalling pathways.

### Knockdown of CD38 alleviated Ang‐II‐induced intracellular Ca^2+^ overload and NFATc4 nucleus expressions

**Figure 5:**
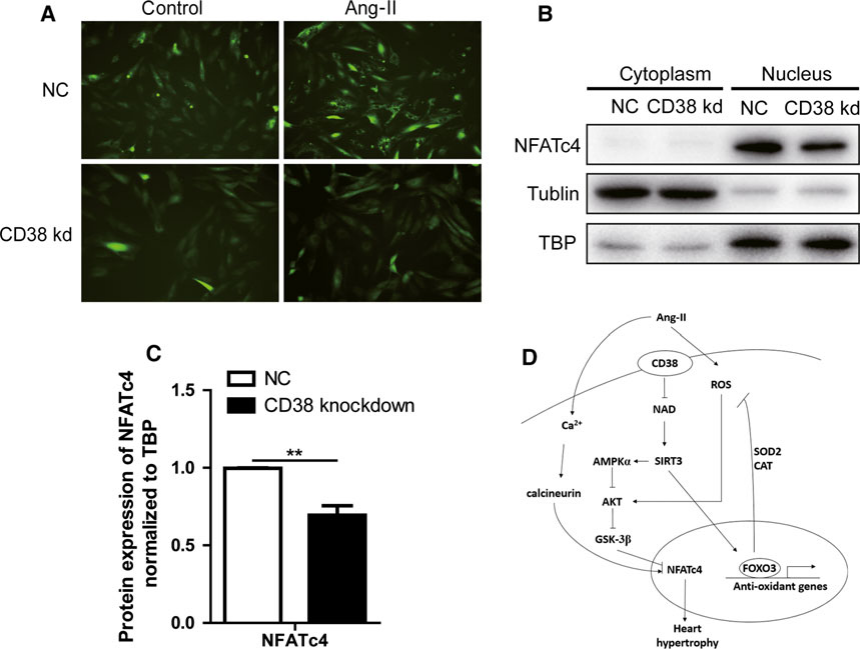
CD38 knockdown attenuated intracellular Ca^2+^ release and inhibited activation of calcineurin–NFATc4 pathway. (**A**) Fluorescent images of intracellular Ca^2+^ in H9c2 cells. The fluorescence was captured using a fluorescent microscope with fluro‐3am in CD38 knockdown or wild‐type H9c2 cells after 20 min. stimulation of Ang‐II (magnification, ×200 scale bars, 50 μm). (**B**) Effects of CD38 on distributions of NFATc4 in H9c2 cells. The contents of NFATc4 protein in cytoplasmic and nuclear fractions were analysed by Western blot in the CD38 knockdown or wild‐type H9c2 cells. TATA‐binding protein (TBP) and tubulin were used as the loading control of nuclear protein or cytoplasmic protein, respectively. (**C**) Quantitative analysis of NFATc4 protein in nucleus. The contents of nuclear NFATc4 protein were quantitatively analysed in the CD38 knockdown or wild‐type H9c2 cells, N = 3, **P < 0.01. (**D**) Graphical abstract for the mechanism of CD38 promoting cardiac hypertrophy. Ang‐II promotes cardiac hypertrophy by activating calcineurin–NFATc4 pathway and enhancing ROS‐mediated Sirt3–FOXO3 pathway. CD38 deficiency protects heart from cardiac hypertrophy through activating Sirt3‐mediated signalling pathway: on the one hand, the increased Sirt3 activity activates AMPKα which inhibits AKT and GSK3β activities, and in turn, promotes phosphorylation of NFATc4, further inhibiting Ang‐II‐mediated calcineurin–NFATc4‐cardiac hypertrophy. On the other hand, the increased Sirt3 activities promote the expressions of some antioxidant genes such as SOD2 and CAT by activating FOXO3 pathway, resulting in attenuation of Ang‐II‐mediated cardiac hypertrophy.

It is well known that the intracellular Ca^2+^ disorder induced by Ang‐II is associated with the development of cardiac hypertrophy, and CD38 as the predominant ADPR‐cyclase is essential for cADPR‐mediated intracellular Ca^2+^ mobilization. Therefore, the intracellular Ca^2+^ was firstly detected with fluro‐3am after Ang‐II stimulation. The result showed that there was a markedly elevation in normal H9c2 cells; however, the intracellular Ca^2+^ in CD38 knockdown cells was much lower than that of control (Fig. 5A).

The NFAT transcription factors are known to play major roles in the induction of cardiomyocyte hypertrophy, and the transcription activity could be regulated by its nucleus‐cytoplasmic shuttling 27. The NFAT transcription factors are exported from the nucleus by phosphorylation, whereas calcineurin, a Ca^2+^‐dependent phosphatase, promotes its nuclear localization by dephosphorylation, enhancing the transcription of genes harbouring NFAT‐binding sites 28. To obtain additional evidence for the effect of CD38 knockdown on the localization of NFATc4, we analysed cytoplasmic and nuclear fractions of cardiomyocytes by Western blotting. The results showed that CD38 knockdown decreased NFATc4 nucleus expressions (Fig. 5B and C). Taken together, our results demonstrated that CD38‐mediated cardiac hypertrophy is also involved in activation of Ca^2+^–calcineurin–NFAT pathway.

## Discussion

Left ventricular hypertrophy is a strong predictor of adverse cardiovascular outcomes and an important risk factor for sudden death and heart failure 29. Many factors could induce ventricular hypertrophy, such as Ang‐II, ET‐1, catecholamines, growth factors and TNF‐α 30. Among them, Ang‐II is commonly used for induction of hypertrophic models, as it can potently induce pressure overload, which leads to an increase in wall thickness and concentric hypertrophy of the left ventricle as a compensatory mechanism to maintain the ventricular EF under conditions of increased peripheral resistance 31. In the present study, we investigated the role of CD38 in the development of Ang‐II‐induced cardiac hypertrophy. Using Ang‐II‐induced hypertrophic models *in vivo* and *in vitro*, we showed that CD38 is a positive regulator of cardiac hypertrophy. The knockout of CD38 gene prevented a hypertrophic response by shutting down the major signalling pathways that induce cardiac hypertrophy. In addition, CD38 knockdown block Ang‐II‐induced oxidative stress by activating the SIRT3‐FOXO3‐mediated antioxidant pathway.

Recent studies have shown that decreased level of oxidative stress is beneficial to alleviating the development of hypertrophy 9, 32. Recently, we found that MEFs from CD38 KO mice were significantly resistant to oxidative stress such as H_2_O_2_‐induced injury and H/R‐induced injury 6 and the CD38 knockout mice protected heart from ischaemia reperfusion injury through activating SIRT1/FOXOs‐mediated antioxidative stress pathway 16. In the present study, the FOXO3–SOD2‐mediated antioxidative effects were significantly enhanced in CD38 knockdown in H9c2 cells, further demonstrating that the protection of CD38 deficiency on cardiac hypertrophy induced by Ang‐II was consistent with our previous findings.

Studies from other laboratories showed that increased level of intracellular NAD^+^ enhanced the activities of SIRT1 and SIRT3 33, which in turn, suppressed cardiac hypertrophy through the activation of Foxo3a‐dependent antioxidant defence mechanism 34. It was also reported that the activation of SIRT3 may promote the expressions of antioxidants including Mn‐SOD 35 and catalase by enhancing the nuclear translocation of FOXO3a 26. CD38 deficiency significantly increased the intracellular contents of NAD^+^ 36 by which the activity of SIRT3 was enhanced. In addition, we also observed that the expressions of SIRT3 were significantly increased in CD38 knockdown H9c2 cell lines with or without Ang‐II stimulation. As expected, the expression of SIRT3 in CD38 knockdown H9c2 cells was much higher than that in negative control cells. However, the mechanism of the up‐regulation of SIRT3 expressions in CD38 knockdown H9c2 cells would be further studied in the future.

Recent years, the roles of sirtuin family in cardiovascular research have attracted increasing attentions. Among all the seven isoforms in mammals, SIRT1, SIRT3, SIRT6 and SIRT7 are all reported to be involved in cardiac growth and their effects vary depending upon experimental conditions. Resveratrol attenuates the phenylephrine‐induced hypertrophic response in cardiomyocytes and pressure overload‐induced cardiac hypertrophy, whereas overexpression of SIRT1 promotes hypertrophy of cardiomyocytes 37. On the other hand, pressure overload co‐up‐regulate SIRT1 and PPAR‐α in the heart, which coordinately suppress genes that are regulated by ERRs, thereby leading to the development of cardiac hypertrophy and heart failure. Thus, the effect of SIRT1 upon cardiac hypertrophy may be affected by a balance of interaction with other factors, such as PPAR‐α, or severity and type of stress 13. However, it was reported that the exogenous NAD^+^ blocks cardiac hypertrophic independent with SIRT1 but SIRT3. Therefore, we focused on the most reported SIRT3, and the protection role was confirmed in our study. The role of SIRT6 in cardiac hypertrophy has been investigated using loss‐of‐function and gain‐of‐function models and manifested to attenuate myocardial hypertrophy by inhibiting NF‐κB activation, AKT signalling pathway and so on 38, 39. SIRT7‐deficient mice showed increased inflammation and inflammatory cardiomyopathy, which was associated with increased pro‐hypertrophic AKT signalling in the heart 40. However, if SIRT6 or SIRT 7 also involved in the protection role of CD38 KO in Ang‐II‐induced hypertrophy still needed further studies.

The ROS‐sensitive MAPK/ERK and PI3K/AKT pathways both have been shown to play a leading role in the development of cardiac hypertrophy by different stimuli, including pressure overload, mechanical stretch and GPCR agonists 3. The growth factor/AKT signalling pathway also promotes cardiac hypertrophy through activation of the mammalian target of rapamycin 41, p70s6k 42 and the inhibition of GSK3β 43. In the present study, we observed that the phosphorylations of ERK and AKT1 were simultaneously attenuated in CD38 knockdown cells after Ang‐II stimulation. In addition, GSK3β, a downstream target of AKT related hypertrophic signalling pathway, was also suppressed in the CD38 knockdown H9c2 cells. Consistent with our results, it has been reported that SIRT3 blocks the cardiac hypertrophic response through activating FOXO‐dependent antioxidants, as well as suppressing ROS‐mediated Ras activation and its downstream signalling pathways such as MAPK/ERK and PI3K/Akt pathways 26. Additionally, it was also reported that the activation of SIRT3–LKB1–AMPK signalling pathway which may further inhibit the pro‐hypertrophic Akt signalling pathway protect hearts from heart hypertrophy 15. Therefore, our study demonstrated that CD38 deficiency‐mediated SIRT3 activation inhibits cardiac hypertrophy through inhibiting the MAPK/ERK and PI3K/Akt signalling pathways which was induced by the ROS decreasing as well as the direct inhibition from SIRT3–LKB1–AMPK signalling pathway.

In the pathological progress of hypertrophy, immune/inflammation was also reported to play an important role, such as IL‐18 which act as a pro‐hypertrophic cytokine through AKT–GATA4 signalling pathway in cardiomyocytes 44, 45, 46. CD38 as an important NAD‐dependent de‐acylation enzyme, it may also play an important role in inflammation regulation through sirtuin‐mediated NF‐κb deacylation. On the hand, CD38 as a widely studied protein in immune system, if it promotes the cardiac hypertrophy through the immune/inflammatory pathways still needs further studies.

It has been reported that exogenous NAD^+^ blocks cardiac hypertrophic response *via* the SIRT3–LKB1–AMP‐activated kinase pathway 15. In skeletal muscle cells, the elevation of intracellular NAD^+^ has been shown to activate AMPKα which attenuated the cardiac hypertrophic response. Moreover, the elevation of NAD^+^ in CD38‐deficient mice may protect mice from cardiac hypertrophy by enhancing some other NAD^+^‐dependent enzymes. It has been reported that the overexpression of NAD^+^‐dependent enzyme ALDH2 rescues chronic alcohol intake‐induced myocardial hypertrophy and contractile dysfunction 47. Furthermore, the alleviation of cardiac hypertrophy by NAD^+^ elevation in CD38 knockout mice is similar to the protection reported in the mutation of another NAD^+^‐consuming enzyme PARP1 48, 49.

It has been shown that IP3‐mediated Ca^2+^ release plays a central role in regulating cardiomyocyte hypertrophy induced by different stimuli, including Ang‐II infusion 50. On the other hand, apart from Ca^2+^ release, Ang‐II receptor activation will also induce Ca^2+^ influx *via* store‐operated Ca^2+^ channels 51. Several recent studied showed that intracellular Ca^2+^ can activate the calcineurin–NFAT pathway and cause pathological cardiac hypertrophy 17, 52. In CD38 knockout mice, there is a significant decrease in the amount of calcium signalling molecule cADPR, suggesting that CD38 plays a role in the Ca^2+^/calcineurin/NFAT signalling pathway. Consistent with our result, Uh‐Hyun Kim *et al*. reported that CD38 KO mice were protected from the ISO‐mediated cardiac hypertrophy *via* Ca^2+^ signalling pathway 53. As we expected, the active NFATc4 expression was significantly decreased in CD38 knockdown cells, suggesting that CD38 deficiency was sufficient to reverse Ang‐II‐induced the activation of the calcineurin–NFATc4 pathway. In addition, a number of potential transcriptional targets of Akt/FOXO signalling in the heart have been identified to be involved in the net loss of cardiac mass. These proteins include the ubiquitination factor E4B, the E3 ubiquitin ligases atrogin‐1/MAFbx and MuRF‐1, which target proteins for proteosomal protein degradation, cathepsin L, a lysosomal proteinase and Igfbp5 54. Recently Li *et al*. demonstrated that atrogin‐1 is expressed in heart and inhibits calcineurin‐dependent cardiac hypertrophy by priming calcineurin for ubiquitin‐mediated proteolysis 55. Therefore, the calcineurin–NFAT signalling pathway might be affected by the change of intracellular Ca^2+^ and even the FOXO3‐atrogin‐1 signalling pathway. However, it needs further studied for validating which one is more important.

## Conclusion

In this study, we are the first to provide the evidence that CD38 deficiency markedly alleviated Ang‐II‐induced cardiac hypertrophy *in vitro* and *in vivo*, through activating the SIRT3‐FOXO3‐mediated anti‐hypertrophic signalling pathway and suppressing the Ca^2+^–calcineurin–NFAT‐mediated prohypertrophic pathway. Certainly, our results should provide an insight in the understanding of the mechanisms of cardiac hypertrophy.

## Competing interests statement

The authors confirm that there are no conflict of interests.

## Supporting information


**Figure S1** The protein expression of CD38 in wildtype and CD38 gene knockout mice.
**Figure S2** Quantitative analysis of %change of collagen contents after Picrosirius red staining.
**Figure S3** Quantitative analysis of %chang of interstitial fibrosis after Masson staining.
