# Supplementary material for: CD38 promotes angiotensin II‐induced cardiac hypertrophy
Source: J Cell Mol Med. 2017 Mar 12;21(8):1492–502. doi: 10.1111/jcmm.13076 (PMC5542907; doi:10.1111/jcmm.13076)

**Figure legends of Supplementary materials:**


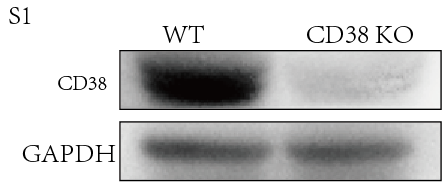
**Figure S1. The protein expression of CD38 in wildtype and CD38 gene knockout mice.** The CD38 expressions were analyzed by Western Blot in wild type or CD38 KO mice, and the result confirmed that there were almost no expression of CD38 in KO mice.

**Figure S2. Quantitative analysis of %change of collagen contents after Picrosirius red staining.** **p*<0.05, ***p*<0.01, ****p*<0.001, N=3


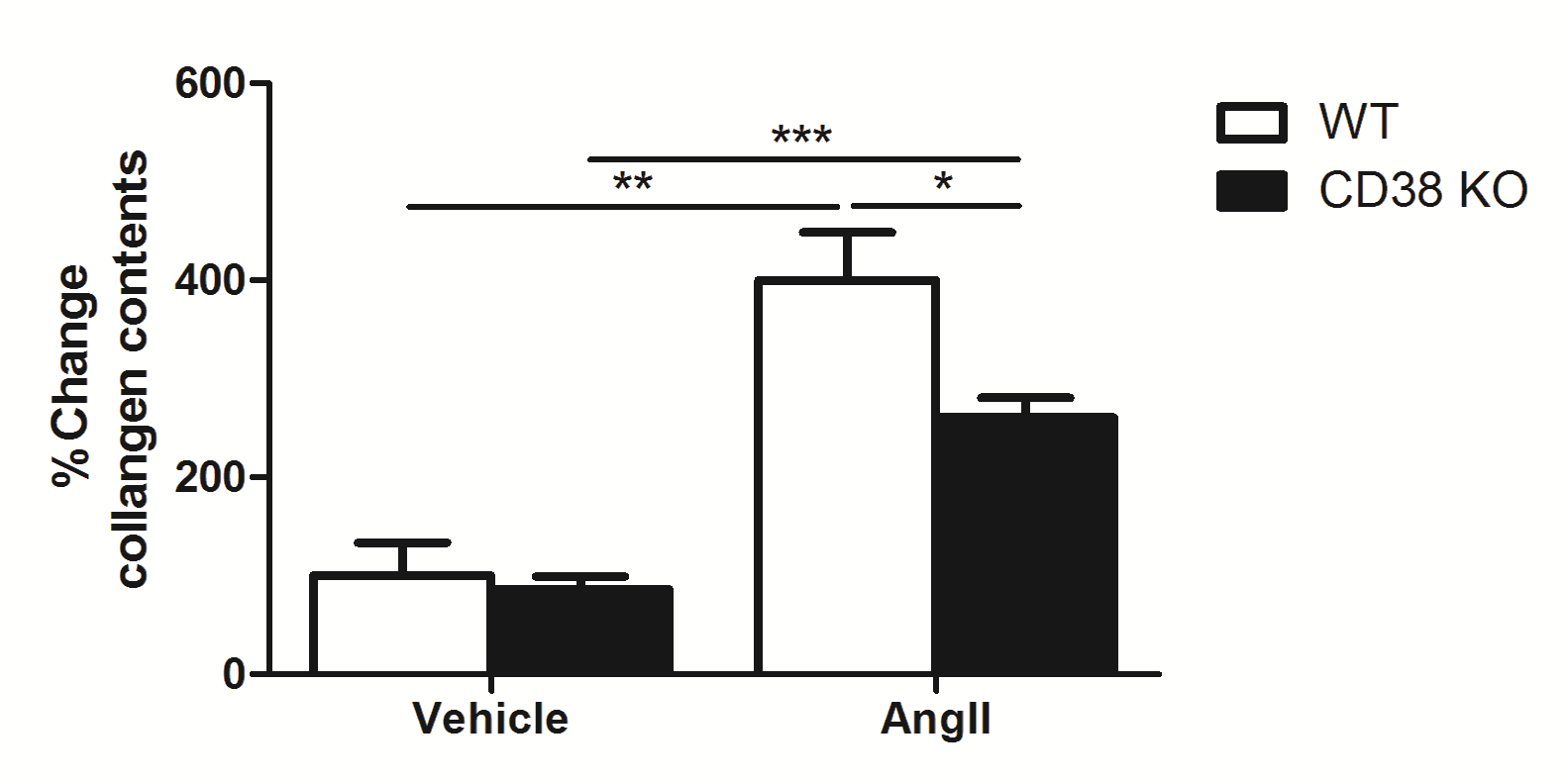


**Figure S3. Quantitative analysis of %chang of interstitial fibrosis after Masson staining.** **p*<0.05, ***p*<0.01, N=3


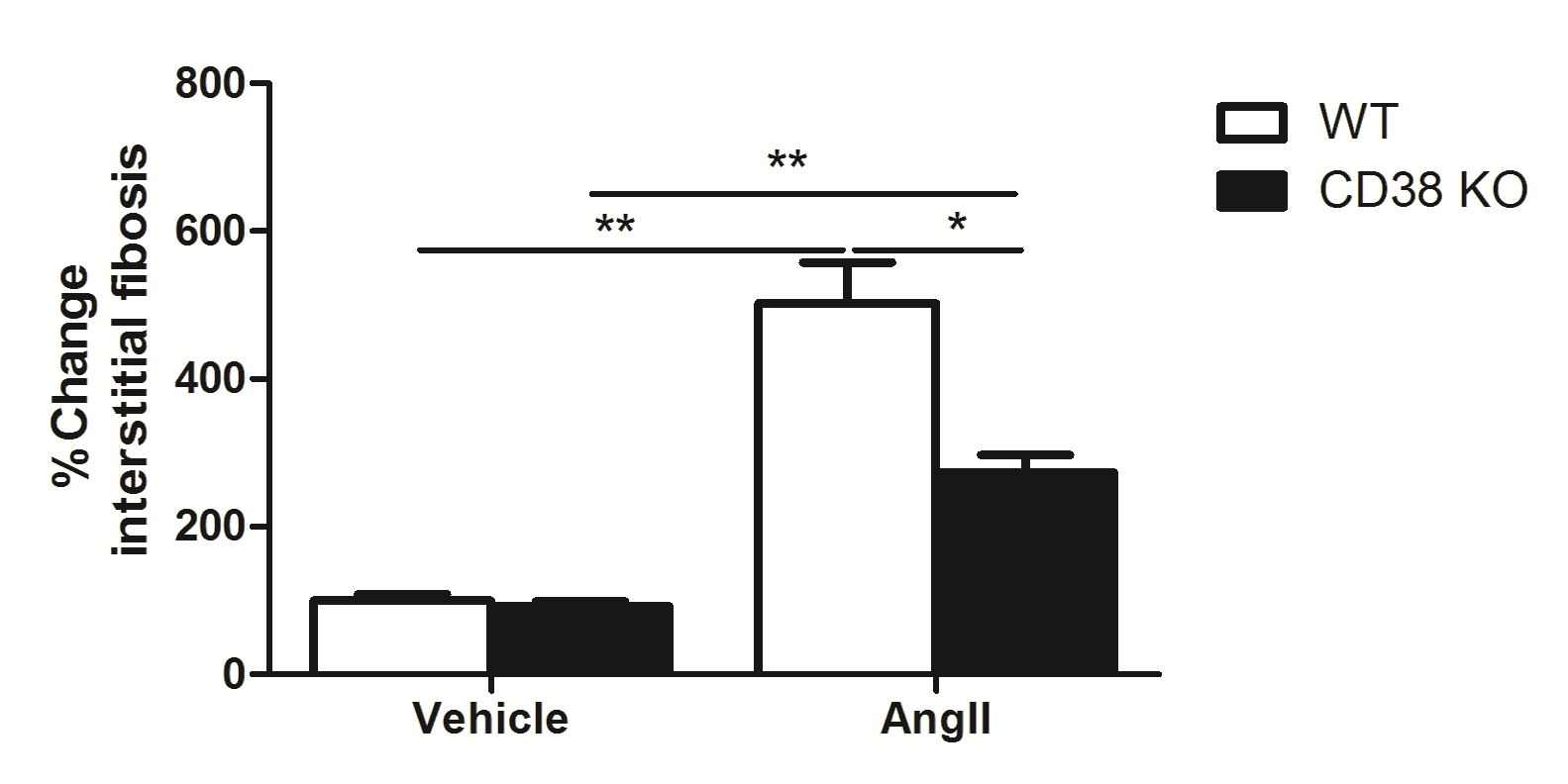

Supplement: Supplementary file 1 — Figure S1 The protein expression of CD38 in wildtype and CD38 gene knockout mice. Figure S2 Quantitative analysis of %change of collagen contents after Picrosirius red staining. Figure S3 Quantitative analysis of %chang of interstitial fibrosis after Masson staining. [file JCMM-21-1492-s001.docx]
